# Special Issue: Frontiers in Antimicrobial Drug Discovery and Design

**DOI:** 10.3390/molecules22071127

**Published:** 2017-07-06

**Authors:** Daniela Barlocco, Fiorella Meneghetti

**Affiliations:** Department of Pharmaceutical Sciences (DISFARM), University of Milan, 20133 Milano, Italy

Since the discovery of Penicillin, antibiotics have saved millions of lives every year. However, the advent of drug resistance has rendered many of them ineffective against several infections caused by organisms such as methicillin-resistant Staphylococcus aureus, Clostridium difficile, multidrug and extensively drug-resistant Mycobacterium tuberculosis and several others. The treatment of infectious diseases is rapidly developing and new effective antibiotics are urgently needed.

In this Special Issue, the search for new molecules has been approached starting both from natural and synthetic compounds and addressing different targets; several strategies were pursued to fight bacterial resistance.

A number of 3-substituted ocotillol-type C-24 epimers, modified from natural 20(*S*)-protopanaxadiol, were synthesized and evaluated for their antibacterial activity by Bi et al. [1]. They were effective both alone and in combination with traditional antibiotics, exhibiting a particularly interesting activity against Gram-positive bacteria and enhancing the susceptibility of *B. subtilis 168* and *Methicillin-Resistant Staphylococcus aureus USA300* to chloramphenicol and kanamycin, making these latter drugs active at much lower concentrations with reduced toxicity. Santos Silva et al. [2] investigated a hydroalcoholic extract of *Himatanthus drasticus* leaves and tested different fractions against several bacterial species. The best activity was shown against *Klebsiella pneumoniae*. The extract, containing, amongst other compounds, plumieride, plumericin or isoplumericin, rutin, quercetin, and chlorogenic acid, was able to inhibit biofilm formation. Special attention was given to bacterial resistance by Riahifard et al. [3] who hypothesized that conjugation or combination of the amphiphilic cyclic peptide [R4W4] with levofloxacin or levofloxacin-Q could improve their antibacterial activity. They employed Fmoc/tBu solid-phase chemistry to synthesize the conjugates. New insight into the Trojan horse strategy for antibiotics design was provided by the valuable results on three heptapeptides: Ran et al. [4] studied the effect of the heptapeptide of Microcin C7 and two analogues, *N*-formylated heptapeptide and *N*-aceylated heptapeptide, on microbial cell growth. Their results showed inhibition of intracellular galactosidase, respiratory chain dehydrogenases, 6-phosphogluconate dehydrogenases and the *Escherichia coli* growth, without destroying the cell membrane integrity. Among the novel validated target for the design of new anti-infective agents, the bacterial sortase A (SrtA), a cysteine transpeptidase that regulates the covalent linkage of several surface protein virulence factors in Gram-positive bacteria, was investigated by Nitulescu et al. [5]. New potential inhibitors were virtually discovered as anti-virulence agents targeting Gram-positive bacteria, including multiresistant strains. The authors employed a computational study, based on structure derived descriptors and molecular fingerprints. Their results indicated the following as the most relevant descriptors for SrtA affinity: the hydrogen acceptor number, the molecular flexibility, molecular weight ranging between 180 and 600, one up to four nitrogen atoms and up to three oxygen atoms.

Particular attention has been given to antitubercolosis and antifungal derivatives. New promising antimycobacterial candidates emerged among a new set of carbohydrate derivatives studied by Korycka-Machala et al. [6]. Starting from the consideration that sugars with heteroatoms other than oxygen have attained considerable importance in glycobiology and in drug design due to their resistance to enzymes such as glycosidases, phosphorylases and glycosyltransferases, the authors screened a set of 21 thio-functionalized carbohydrate derivatives against acid-fast *Mycobacterium tuberculosis* (Mtb) and several other bacteria. Tseng et al. [7] investigated a series of indeno[1,2-*c*]quinoline compounds as dual anti-TB and anti-inflammatory drug candidates. Amongst them, (*E*)-*N*′-[6-(4-hydroxypiperidin-1-yl)-11*H*-indeno[1,2-*c*]quinolin-11-ylidene]isonicotino-hydrazide exhibited significant activity against the growth of Mtb (MIC values of 0.96 μg/mL), with a potency approximately equal to that of isoniazid (INH), accompanied by interesting anti-inflammatory properties.

As a leading synthetic structure for the development of novel fungicides, the benzimidazole phenylhydrazone scaffold was exploited. A number of its derivatives, investigated by Wang et al. [8] exhibited significant activity against the phytopathogenic fungi, *Rhizoctonia solani* and *Magnaporthe oryzae*. Moreover, Wang et al. [9] developed a series of thirty-eight sulfonyl derivatives, which were tested in vitro and in vivo against two *Botrytis cinerea* strains, DL-11 and HLD-15, in comparison with procymidone. Most of them showed better effects than the reference drug. Additionally, natural products have played a fundamental role in the development of new therapeutic agents. Su et al. [10] exploited essential oil from plant-derived aroma compounds to synthesize a series of methoxyacrylate derivatives which have been screened against eleven species of plant pathogen fungi, including *Alternaria alternata, Phomopsis adianticola, Pestalotiopsis theae,* and *Sclerotinia sclerotiorum*. Almost all of them exhibited significantly better fungicidal activity than the parent natural oils and azoxystrobin, a well-known antifungal agent.

High bactericidal and anti-fungal activities were also displayed by twelve new synthetic analogs of α-mangostin from *Garcinia mangostana,* investigated by Narasimhan et al. [11], who suggested that they could be utilized both for biomedical materials and food industry. Zoric et al. [12] reported that Oleuropein, a complex phenol present in large quantities in olive tree products, was active against the opportunistic fungal pathogen *Candida albicans*; the morphogenetic conversion and filamentation inhibition modulated by oleuropein was described, together with the reduction in total sterol content in the cell membranes, which is also involved in the mechanism of its antifungal activity. The authors concluded that it causes cell death mainly through apoptosis.

Finally, viruses are as dangerous as bacteria. Starting from curcumin, Yu et al. [13] designed a series of novel 1,4-pentadien-3-one derivatives containing a 1,3,4-thiadiazole moiety. They showed remarkable inhibitory activity in vivo against plant viruses, e.g., tobacco mosaic virus (TMV) and cucumber mosaic virus. Preliminary SARs indicated that small electron-withdrawing groups on the aromatic ring were favorable for anti-TMV activity.
